# Assembly of α-synuclein and neurodegeneration in the central nervous system of heterozygous M83 mice following the peripheral administration of α-synuclein seeds

**DOI:** 10.1186/s40478-021-01291-7

**Published:** 2021-11-24

**Authors:** Jennifer A. Macdonald, John L. Chen, Masami Masuda-Suzukake, Manuel Schweighauser, Zane Jaunmuktane, Thomas Warner, Janice L. Holton, Annabelle Grossman, Richard Berks, Isabelle Lavenir, Michel Goedert

**Affiliations:** 1 MRC Laboratory of Molecular Biology, Francis Crick Avenue, Cambridge, CB2 0QH UK; 2 Queen Square Brain Bank for Neurological Disorders, UCL Institute of Neurology, 1 Wakefield Street, London, WC1N 1PJ UK; 3 Kymab, Babraham Research Campus, Cambridge, CB22 3AT UK

**Keywords:** α-Synuclein, Multiple system atrophy, Seeded assembly, Neurodegeneration, Microglia

## Abstract

Peripheral administration (oral, intranasal, intraperitoneal, intravenous) of assembled A53T α-synuclein induced synucleinopathy in heterozygous mice transgenic for human mutant A53T α-synuclein (line M83). The same was the case when cerebellar extracts from a case of multiple system atrophy with type II α-synuclein filaments were administered intraperitoneally, intravenously or intramuscularly. We observed abundant immunoreactivity for pS129 α-synuclein in nerve cells and severe motor impairment, resulting in hindlimb paralysis and shortened lifespan. Filaments immunoreactive for pS129 α-synuclein were in evidence. A 70% loss of motor neurons was present five months after an intraperitoneal injection of assembled A53T α-synuclein or cerebellar extract with type II α-synuclein filaments from an individual with a neuropathologically confirmed diagnosis of multiple system atrophy. Microglial cells changed from a predominantly ramified to a dystrophic appearance. Taken together, these findings establish a close relationship between the formation of α-synuclein inclusions in nerve cells and neurodegeneration, accompanied by a shift in microglial cell morphology. Propagation of α-synuclein inclusions depended on the characteristics of both seeds and transgenically expressed protein.

**Supplementary Information:**

The online version contains supplementary material available at 10.1186/s40478-021-01291-7.

## Introduction

The ordered assembly of a small number of proteins into pathological amyloid filaments defines most human neurodegenerative diseases, including Alzheimer’s (AD) and Parkinson’s (PD) [1]. α-Synuclein assemblies are characteristic of PD, PD dementia, dementia with Lewy bodies (DLB), multiple system atrophy (MSA) and several rarer conditions, collectively referred to as synucleinopathies [2]. In these diseases, the 140 amino acid α-synuclein assembles into a filamentous, β-sheet-rich conformation. Unbranched α-synuclein filaments are 5–10 nm in diameter and up to several micrometres long, mostly in nerve cells (Lewy bodies and neurites) and, for MSA, glial cells, chiefly oligodendrocytes (Papp-Lantos bodies).

A link between α-synuclein assembly and disease was established by the findings that missense mutations in *SNCA* (the α-synuclein gene), and multiplications thereof, cause rare forms of PD and PD dementia [3, 4]. Some mutations and multiplications also give rise to DLB. Abundant α-synuclein inclusions are present in all cases of inherited disease. Sequence variations in the regulatory region of *SNCA* are associated with increased α-synuclein expression and a heightened risk of sporadic PD [5].

Accumulating evidence indicates that assembled α-synuclein propagates in the nervous system in a manner akin to prions. *Post mortem* staging shows progression of α-synuclein pathology in a defined spatiotemporal pattern, resulting in PD and DLB [6]. Assembled α-synuclein first appears in the dorsal motor nucleus of the vagus nerve in the brainstem, before ascending to anatomically related regions in midbrain and cerebral cortex. Additional evidence comes from the apparent spreading of Lewy pathology from host to graft, following transplantation of foetal human mesencephalic tissues as a treatment for PD [7, 8]. These findings are extended by transmission studies, where assembled α-synuclein, in the form of aggregated recombinant protein or brain-derived material, is injected intracerebrally [9–12] or peripherally [13–24]. One study described the effects of oral administration of assembled recombinant α-synuclein [25].

The dual-hit hypothesis of PD posits that the filamentous assembly of α-synuclein begins in the nose and the digestive tract, following entry of a pathogen through the nasal cavity, which can reach the gut following swallowing [26]. In support, PD patients often report deficits in gastrointestinal motility and olfaction early in the disease process [27]. Full truncal vagotomy has been reported to lower the risk of developing PD by 40–50% after 10–20 years of follow-up [28, 29].

Here we investigated the effects of oral, nasal, intravenous, intraperitoneal and intramuscular administration of α-synuclein seeds on assembly, neurodegeneration and neuroinflammation. Following the oral and nasal administrations, implicated in the dual-hit hypothesis, we characterised for the first time the initial sites affected by α-synuclein pathology and their similarity to those first implicated in prion transmission [30, 31]. We used 3-month-old heterozygous mice transgenic for human A53T α-synuclein (line M83) [32], that do not develop synucleinopathy until at least 20 months of age. Irrespective of the mode of administration, we observed synucleinopathy after less than 10 months, as evidenced by the formation of abundant filamentous α-synuclein inclusions and a severe impairment of motor behaviour. For the first time, we assessed neurodegeneration in relation to α-synuclein pathology by quantitating the number of motor neurons and pS129 α-synuclein immunoreactivity in spinal cord following intraperitoneal injection of assembled recombinant human A53T α-synuclein or cerebellar extract from a case with type II MSA filaments, as determined by electron cryo-microscopy (cryo-EM), thereby establishing a direct correlation between a specific MSA filament type and neurodegeneration. Finally, we analysed microglial cell morphology over time in animals injected intraperitoneally with assembled α-synuclein.

## Materials and methods

### Expression, purification and assembly of A53T α-synuclein

Human A53T α-synuclein or human A53T α-synuclein lacking residues 71–82 (Δ71–82), which shows a greatly reduced ability to assemble into filaments [33], was expressed as described [34, 35]. Bacterial pellets were resuspended in 10 mM Tris–HCl, pH 7.4, containing protease inhibitor tablets (Roche), sonicated using a Vibracell ultrasonic probe (4 min, alternating between 5s ON and 5s OFF, at an amplitude of 40%) and centrifuged for 20 min at 20,000 rpm. Supernatants were passed through a 0.45 μm filter and loaded onto a HiTrap Q HP ion exchange column. Fractions were eluted using an increasing gradient of elution buffer (10 mM Tris–HCl, pH 7.4, 1 M NaCl). Following SDS-PAGE, they were precipitated with 250 μg/ml ammonium sulphate for 30 min at 4° C and centrifuged for 20 min at 20,000 rpm. Pellets were stored at −80° C. Following resuspension in 50 mM Tris–HCl, pH 7.4, 150 mM NaCl and a 15 min centrifugation at 15,000 rpm, the supernatants were loaded onto a HiLoad 16/600 Superdex 75 pg gel filtration column and eluted with 50 mM Tris–HCl, 150 mM NaCl. Following SDS-PAGE and ammonium sulphate precipitation, the pellets were resuspended in PBS and dialysed overnight. Following a 30 min centrifugation at 45,000 rpm, supernatants were collected and stored at − 80 °C. Concentrations of purified α-synuclein were determined using a NanoDrop spectrophotometer (Thermo Fisher). Assembly of recombinant α-synuclein was performed as described [35]. The assemblies were sonicated using an XL2020 ultrasonic processor (Misonix) at output level 2 (ON, 0.9 s, OFF 0.1 s, for a total of 5 s). The average filament length was 57 ± 2 nm (n = 200).

### Administration of assembled A53T α-synuclein

Experiments used 3-month-old heterozygous M83 mice. For nasal administration, mice received 50 μl of 400 μM assembled A53T α-synuclein (0.28 mg) over both nostrils daily for 28 days. Oral administration was achieved by daily gavage using 20-gauge plastic tubes (Instech Laboratories) of 200 μl of 400 μM assembled A53T α-synuclein (1.1 mg) for 28 days. Intravenous injection consisted of daily injections of 1 mg assembled A53T α-synuclein into the tail vein for 4 days. In some experiments, 100 μg, 10 μg or 1 μg were injected. For intraperitoneal injection, 200 μl of 400 μM assembled A53T α-synuclein (1.1 mg) or assembled Δ71–82 A53T α-synuclein was administered. PBS was used as control. Motor impairment was assessed by Rotarod, using acceleration from 4 to 40 rpm over 5 min. The time was recorded when mice fell from the rod or when they rotated passively for two consecutive revolutions. Severe motor impairment consisted of abnormal gait, abnormal posture when lifted by the tail, hindlimb paralysis and an abnormal righting reflex.

### Multiple system atrophy

Cerebellum from a 68-year-old male with a neuropathologically confirmed diagnosis of MSA was homogenised in PBS at 100 mg/ml or 200 mg/ml. At autopsy, numerous α-synuclein-positive inclusions were present in motor cortex, striatum, substantia nigra, pontine nuclei, inferior olive and cerebellum. They were glial cytoplasmic inclusions and, for some regions, also neuronal cytoplasmic and intranuclear inclusions. Homogenates were sonicated using an XL2020 ultrasonic processor (Misonix) at output level 2 (ON, 0.9 s, OFF 0.1 s, for a total of 5 s). Following a 5 min centrifugation at 3,000 g, the supernatants were aliquoted and stored at -80° C until use. Injections were given intravenously, intramuscularly or intraperitoneally. As the control, cerebellum from a 68-year-old male without synucleinopathy was used at 200 mg/ml.

### Antibodies

To detect assembled α-synuclein by immunohistochemistry, we used two anti-pS129 α-synuclein antibodies (clone 64, Wako and EP1536Y, Abcam) and two anti- α-synuclein antibodies (1903, Abcam and LB509, Covance). Phosphorylation-dependent antibodies were used at 1:1,000 and phosphorylation-independent antibodies at 1:10,000. To assess microglia in the context of α-synuclein inclusions, anti-ionized calcium binding adaptor molecule 1 (Iba1) (019–9741, 1:500, Wako), a panel of epitope-specific anti-α-synuclein antibodies (α-Syn34-45, 1:200, BioLegend; α-Syn80-96, 1:100, BioLegend; α-Syn117-122, 1:100, BioLegend) and an antibody specific for α-synuclein phosphorylated at S129 (EP1536Y, 1:750 or ab184674 1:500, Abcam) were used. For negative-stain immunoelectron microscopy, we used ab59264 and PER4 at 1:100 or ab51253 at 1:50 [35]. For immunoblotting, antibodies were also used at 1:1,000. They included two mouse anti- α-synuclein antibodies (Syn 1, BD Biosciences; Syn211, Santa Cruz), as well as ab59264 and ab51253. To quantify motor neuron loss, an anti-NeuN antibody (Millipore) was used at 1:500.

### Immunohistochemistry

Mice were perfused transcardially with 4% paraformaldehyde in 0.1 M PBS, pH 7.4. Brains and spinal cords were dissected and post-fixed overnight at 4°C, followed by cryo-protection in 20% sucrose in PBS for a minimum of 24 h. Coronal brain sections (40 μm) were cut using a VT1000 S vibratome (Leica). Transverse spinal cord sections (30 μm) were cut on a Leica SM2400 microtome (Leica Microsystems). Sections were stored at 4° C in PBS containing 0.1% sodium azide. Endogenous peroxidase activity was quenched by incubation in 0.3% H_2_O_2_ for 30 min. Following a brief wash in PBS + 0.1% Triton-X100 (PBST), the sections were incubated in blocking buffer (PBST + 5% normal goat serum) for 15 min. This was followed by an overnight incubation at room temperature with primary antibody in blocking buffer. After three rinses with PBST, the sections were incubated with biotin-conjugated secondary antibodies for 1 h at room temperature. Following a further three rinses with PBST, the avidin–biotin-conjugated complex was applied at room temperature for 30 min. The signal was visualised with the Vector VIP substrate kit (Vector Laboratories). Tissue sections were mounted on frosted end glass slides (Thermo Scientific) and coverslipped.

### Fluorescence

Free-floating brain sections were incubated for 1 h in 5% normal goat serum in PBST. For epitope-specific α-synuclein antibodies, tissue sections were first treated with 80% formic acid for 1 min at room temperature prior to blocking. Formic acid was not used for antibodies specific for α-synuclein phosphorylated at S129. Sections were incubated in primary antibodies overnight at 4° C. Secondary antibodies conjugated to Alexa Fluor 488 or 594 were then added and incubated for 2 h at room temperature. Prior to labelling with DAPI, some sections were incubated with 3 μM pentameric formyl thiophene acetic acid (pFTAA) for 30 min, followed by 3 washes in PBST. Autofluorescence was quenched by immersing sections for 30 s in TrueBlack Lipofuscin Autofluorescence Quencher (Biotium) solution, followed by 3 washes in distilled water. Sections were then mounted on SuperFrost slides (Thermo Fisher) and coverslipped with Vectashield Antifade mounting medium (Vector Labs). Images were acquired on an LSM 780 confocal microscope (Zeiss).

### Sarkosyl extraction and immunoblotting

Brains and spinal cords were homogenised at 10 ml/g in 10 mM Tris–HCl, pH 7.4, 800 mM NaCl, 10% sucrose, 1 mM EGTA and 1 mM PMSF with proteinase inhibitors. The sarkosyl-insoluble fraction was prepared as described [36]. Pellets were resuspended in 150 μl/g tissue of 50 mM Tris–HCl, pH 7.4, and stored at 4°C. For immunoblotting, samples were run on Novex 8% or 10% Tris–Glycine gels (Thermo Fisher) and transferred onto nitrocellulose membranes. Blots were incubated overnight with primary antibodies, followed by either anti-mouse or anti-rabbit HRP-conjugated secondary antibodies, and the signal was visualised by enhanced chemiluminescence (GE Healthcare).

### Immunoelectron microscopy

Immunoelectron microscopy was done as described [37]. Syn211 (Covance), Syn303 (Covance) and anti-pS129 α-synuclein (Abcam) were used at 1:50. Images were acquired at 11,000 × using a Tecnai G2 Spirit transmission electron microscope at 120 kV.

### Electron cryo-microscopy

α-Synuclein filaments were extracted from the cerebellum of a neuropathologically confirmed case of MSA, as described [38], placed on glow-discharged holey carbon gold grids (Quantifoil R1.2/1.3, 300 mesh) and plunge-frozen in liquid ethane using an FEI Vitrobot Mark IV. Micrographs were acquired using a Gatan K2 summit detector in counting mode on a Titan Krios microscope (Thermo Fisher) at 300 kV. Reference-free 2D classification and cryo-EM maps were obtained as described [38]. For assembled tau from Alzheimer’s disease brain, we have shown that the use of sarkosyl during extraction does not affect the structures of tau filaments [39].

### Stereology

Stereology was carried out as described [40]. Briefly, we counted from 8 randomly chosen sections out of 96 sections/L3-L5 spinal cord. Section thickness was determined using StereoInvestigator 11 software. For each section, the outline of the region of interest was traced under an × 5 objective, starting from the middle of the central canal and contouring the grey matter of the ventral horn. Using the optical fractionator probe (grid size: 65 × 65 μm^2^; height: 22 μm; guard height: 3 μm; counting frame: 50 × 50 μm^2^), NeuN-positive cells, with a diameter of at least 30 μm and with their nuclei in the dissector volume, were counted using the × 100 objective, and the number of motor neurons per lumbar spinal cord calculated. The investigator was blinded with respect to the nature of the two groups.

### Image quantitation

Photographs were taken using an Olympus BX41 microscope equipped with a Nikon digital Sight DS-2Mv digital camera under a × 20 objective. Each picture amounted to 1,600 × 1,200 pixels. Immunoreactivity for pS129- α-synuclein was quantitated using the green channel of ImageJ. The threshold was set to 110 in greyscale. The circularity was 10-infinity. For assessment of microglial morphology, the investigator annotated Iba1-immunoreactive microglia based on morphology from photographs taken under a × 20 objective. The investigator was blinded with respect to the nature of the groups.

### Statistics

Analyses were carried out using GraphPad Prism 7 software. They included log-rank tests for survival data and unpaired one-tailed t-tests for rotarod data and electron micrograph measurements. The significance level was set at *p* < 0.05, with the Bonferroni correction for multiple comparisons. For cell counting, a two-way ANOVA, followed by Dunnett’s or Tukey’s multiple comparisons test and a one-way ANOVA, followed by Tukey’s multiple comparisons test, were used.

## Results

### Seeded aggregation and motor impairment following oral and nasal administration of assembled A53T α-synuclein

**Fig. 1:**
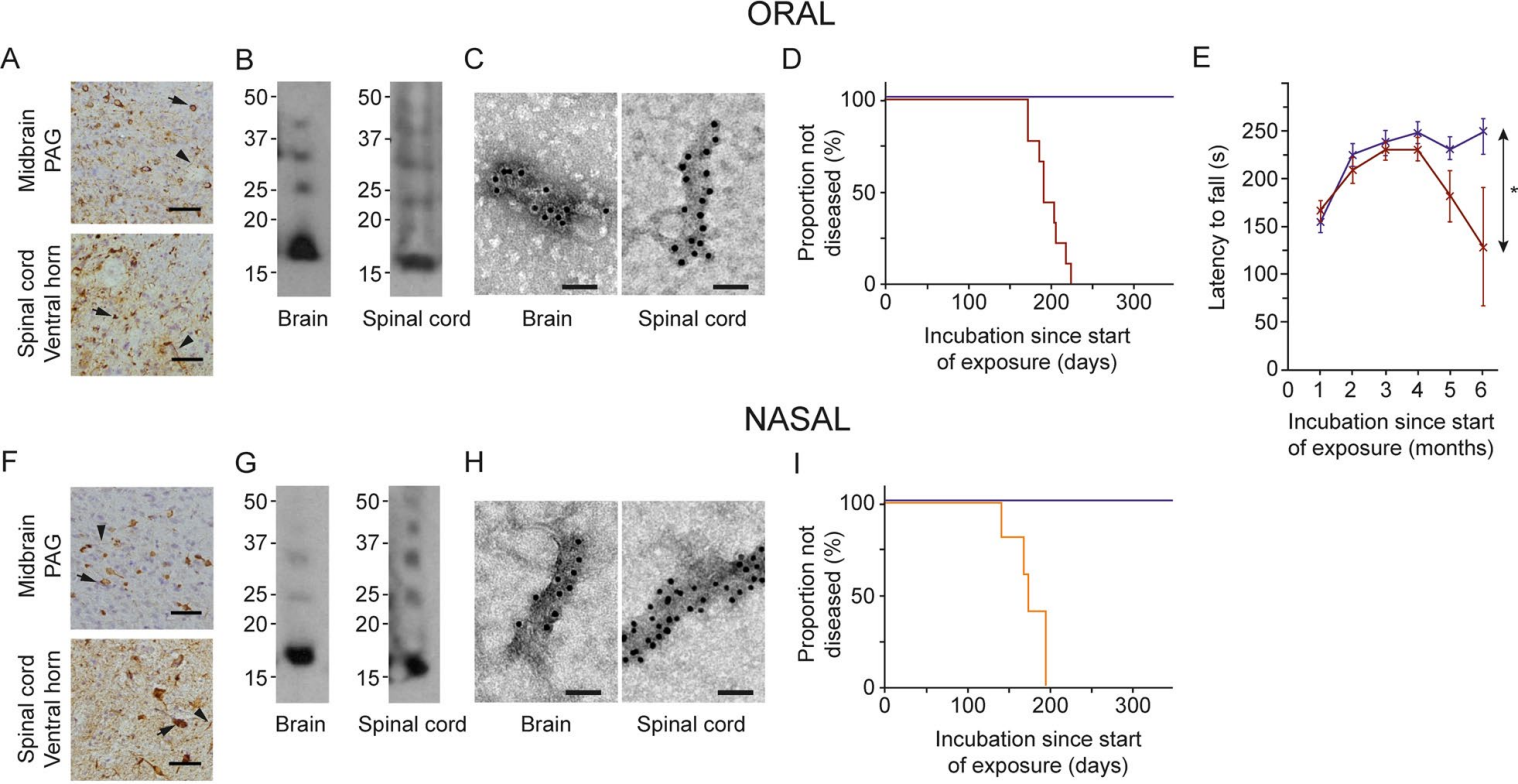
Synucleinopathy in M83^±^ mice following oral (**A**–**E**) and nasal (**F**–**I**) administration of assembled A53T α-synuclein. **A** Staining of midbrain periaqueductal grey (PAG) and lumbar spinal cord (ventral horn) for pS129 α-synuclein (Wako pSyn#64). Arrows denote perikaryal and arrowheads neuritic staining. Scale bars, 50 μm. **B** Immunoblots of sarkosyl-insoluble material from brain and spinal cord using an antibody specific for pS129 α-synuclein (Abcam ab51253). **C** Negative-stain immunoelectron microscopy of filaments from brain and spinal cord using an antibody specific for pS129 α-synuclein (Abcam ab51253). Scale bars, 50 nm. **D** Kaplan–Meier survival curves following oral administration of assembled A53T α-synuclein (red) or PBS (purple). **E** Motor impairment on rotarod testing following oral administration of assembled A53T α-synuclein (red) compared to controls (purple). Unpaired one-tailed t-test for 6-month time-point. *p = 0.03. **F** Staining of midbrain periaqueductal grey (PAG) and lumbar spinal cord (ventral horn) for pS129 α-synuclein (Wako pSyn#64). Arrows denote perikaryal and arrowheads neuritic staining. Scale bars, 50 μm. **G** Immunoblots of sarkosyl-insoluble material from brain and spinal cord using an antibody specific for pS129 α-synuclein (Abcam ab51253). **H** Negative-stain immunoelectron microscopy of filaments from brain and spinal cord using an antibody specific for pS129 α-synuclein (Abcam ab51253). Scale bars, 50 nm. **I** Kaplan–Meier survival curves following nasal administration of assembled A53T α-synuclein (orange) or PBS (purple)

Oral administration consisted in daily gavaging of heterozygous mice transgenic for human A53T α-synuclein (line M83) with 200 μl of 400 μM assembled A53T α-synuclein for 28 days. Nasal administration involved daily administration of 50 μl of 400 μM assembled A53T α-synuclein for 28 days. We used an antibody specific for α-synuclein phosphorylated at S129 to show widespread staining throughout brain and spinal cord 5 months after oral and nasal administration of assembled A53T α-synuclein (Fig. 1A,F). Assembled α-synuclein is phosphorylated at S129 in human brain [41, 42]. Brains and spinal cords were extracted 5 months after seed administration using sarkosyl. Western blotting of the sarkosyl-insoluble fraction showed α-synuclein bands of approximately 15 kDa (Fig. 1B,G). By immunoelectron microscopy, α-synuclein filaments were present (Fig. 1C,H).

Following the administration of assembled A53T α-synuclein by either the oral or the nasal route, M83^±^ mice developed severe motor abnormalities characterised by abnormal posture and gait, hindlimb dysfunction, inability to right and, eventually, paralysis. Mean time from aggregate delivery to end stage disease was 195 ± 6 days (n = 10) after oral (Fig. 1D) and 170 ± 10 days (n = 6) following nasal (Fig. 1I) administration. Significant motor impairment was in evidence upon rotarod testing of mice 6 months after oral administration of assembled A53T α-synuclein (Fig. 1E). When PBS was administered, no abnormalities were observed.

**Fig. 2:**
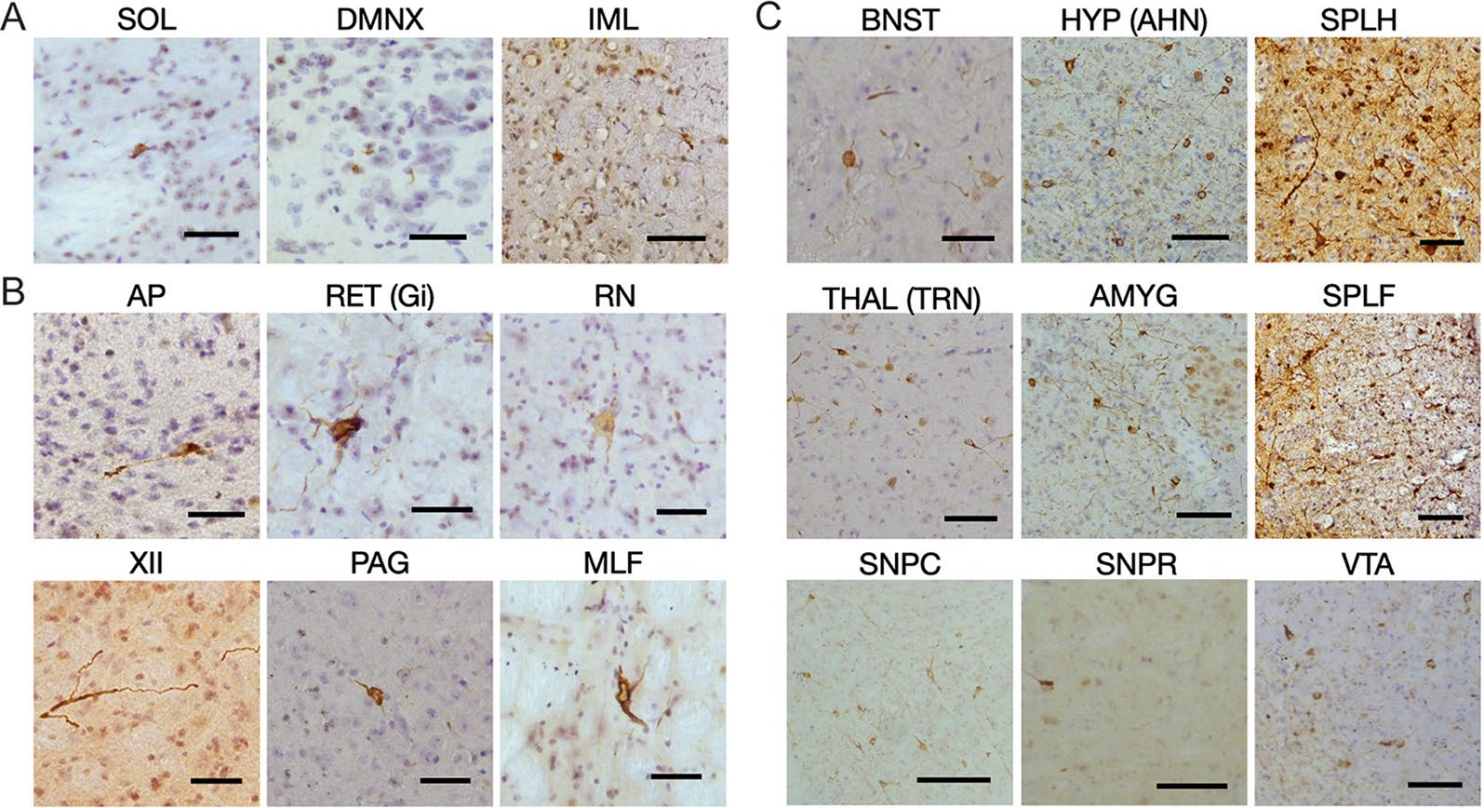
Staining for pS129 α-synuclein (Wako pSyn#64) following oral administration of assembled A53T α-synuclein to M83^±^ mice. **A** Two months after oral administration. SOL, solitary nucleus; DMNX, dorsal motor nucleus of the vagus nerve; IML, intermediolateral nucleus of the thoracic spinal cord. **B** Additional staining three and four months after oral administration. AP, area postrema; RET, reticular formation (gigantocellular nucleus, Gi); RN, raphe nucleus; XII, hypoglossal nucleus; PAG, periaqueductal grey; MLF, medial longitudinal fasciculus. **C** Additional staining five months after oral administration until endpoint. BNST, bed nucleus of the stria terminalis; HYP, hypothalamus (anterior nucleus, AHN); SPLH, lateral horn of the spinal cord; THAL, thalamus (reticular nucleus, TRN); AMYG, amygdala; SPLF, spinal lateral funiculus, SNPC, substantia nigra pars compacta; SNPR, substantia nigra pars reticulata; VTA, ventral tegmental area. Scale bars, 50 μm

Staining for pS129 α-synuclein following oral administration of seeds was present after 2 months in the nucleus of the solitary tract, dorsal motor nucleus of the vagus nerve and intermediolateral spinal cord (Fig. 2A). After 3–4 months, staining also appeared in the area postrema, hypoglossal nucleus, spinal trigeminal nucleus, reticular formation, raphe nuclei, inferior olive, thalamus, periaqueductal grey, superior and inferior colliculi, medial longitudinal fasciculus and spinal cord white matter (Fig. 2B). The regions most affected at 5 months were the thalamus, hypothalamus, amygdala, basal forebrain, stria terminalis, periaqueductal grey, reticular formation and spinal cord (Fig. 2C). Small numbers of α-synuclein inclusions were also seen in pars compacta of the substantia nigra and ventral tegmental area.

### Seeded aggregation and motor impairment following intravenous injection of assembled A53T α-synuclein

**Fig. 3:**
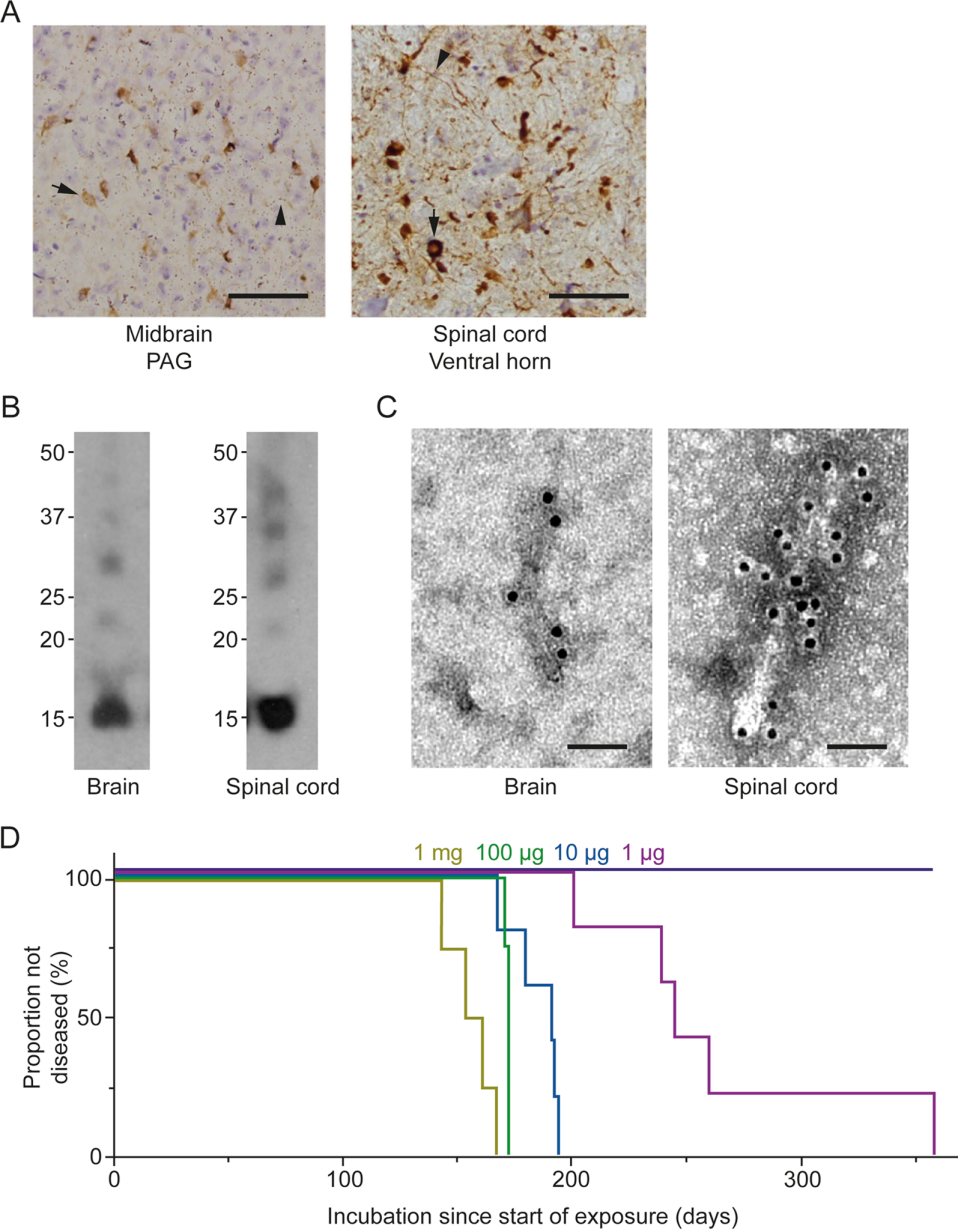
Synucleinopathy in M83^±^ mice following intravenous injection of assembled A53T α-synuclein. **A** Staining of midbrain periaqueductal grey (PAG) and lumbar spinal cord (ventral horn) for pS129 α-synuclein (Wako pSyn#64). Arrows denote perikaryal and arrowheads neuritic staining. Scale bar, 50 μm. **B** Immunoblots of sarkosyl-insoluble material from brain and spinal cord using an antibody specific for pS129 α-synuclein (Abcam ab51253). **C** Negative-stain immunoelectron microscopy of α-synuclein filaments from brain and spinal cord using an antibody specific for pS129 α-synuclein (Abcam ab51253). Scale bar, 50 nm. **D** Kaplan–Meier survival curves following intravenous injection of different amounts of assembled A53T α-synuclein or PBS. Gold, 1 mg; green, 100 μg; blue, 10 μg; magenta, 1 μg; purple, PBS

M83^±^ mice received 1 mg assembled A53T α-synuclein intravenously on 4 consecutive days. They developed synucleinopathy and progressive motor dysfunction and were culled after 156 ± 5 days (n = 4) (Fig. 3). Injections of smaller amounts of assembled A53T α-synuclein (100 μg, 10 μg, 1 μg) also resulted in motor dysfunction, but with longer incubation periods (Fig. 3D).

### Motor neuron loss correlates with increased pS129 α-synuclein immunoreactivity after intraperitoneal injection of assembled A53T α-synuclein

**Fig. 4:**
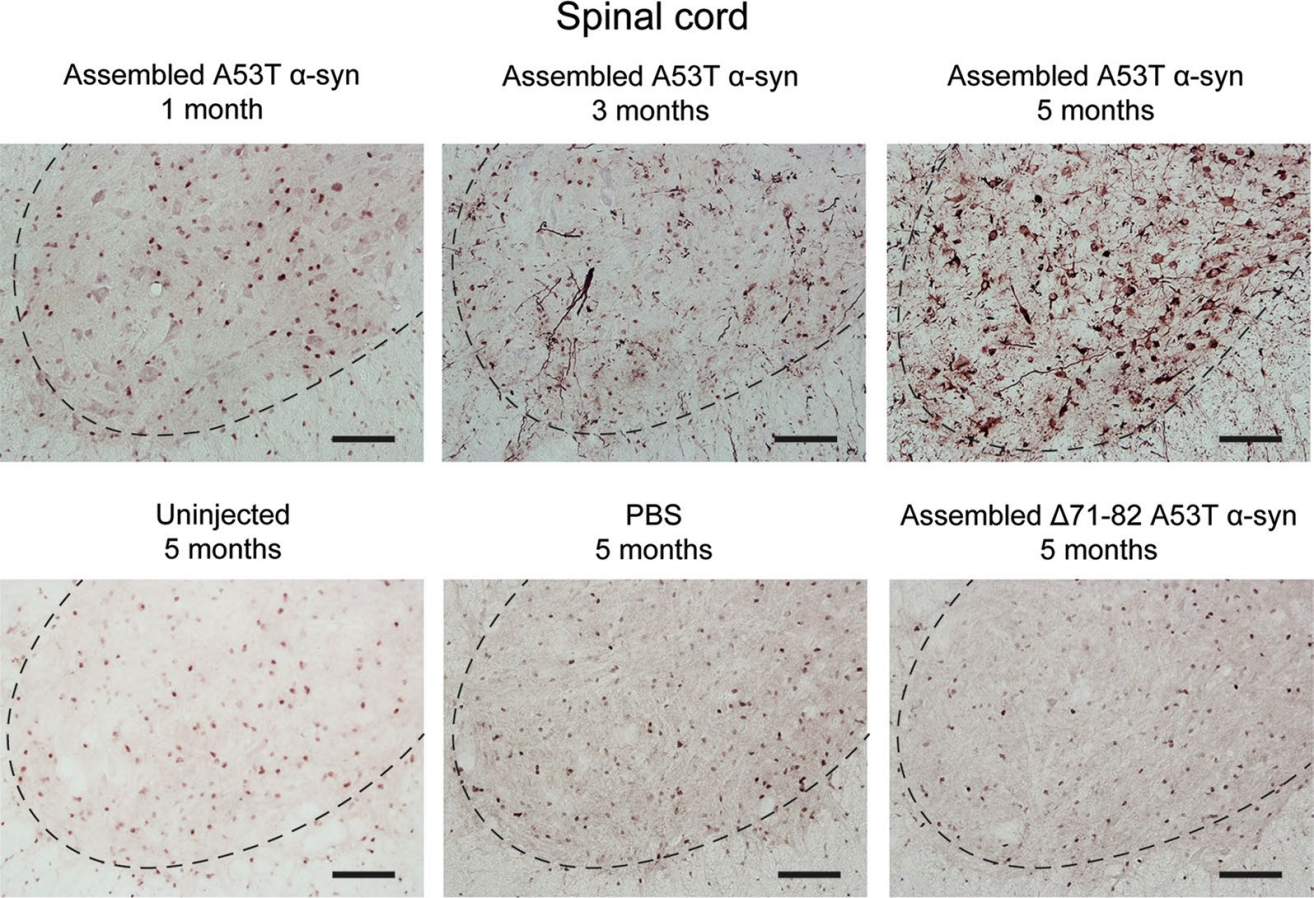
Staining of lumbar spinal cord from M83^±^ mice for pS129 α-synuclein (Abcam, EP1536Y) following intraperitoneal injection of assembled A53T α-synuclein. Staining was in evidence 3 and 5 months, but not 1 month, after injection. No specific staining was seen after 5 months in uninjected, PBS-injected or M83^±^ mice injected with assembled Δ71–82 A53T α-synuclein. The dashed line delineates the ventral horn. Scale bars, 100 μm

M83^±^ mice received an intraperitoneal injection of 200 μl of 400 μM assembled A53T α-synuclein. As shown in Fig. 4, no additional staining for pS129 α-synuclein was observed one month after injection. At 3 months, the difference between mice injected with assembled A53T α-synuclein and PBS was 30%. It was 1200% at 5 months (Additional file 1: Figure 1). In mice injected with assembled Δ71–82 A53T α-synuclein, immunoreactivity for pS129 α-synuclein was not significantly different from that of mice injected with PBS or of uninjected mice.

**Fig. 5:**
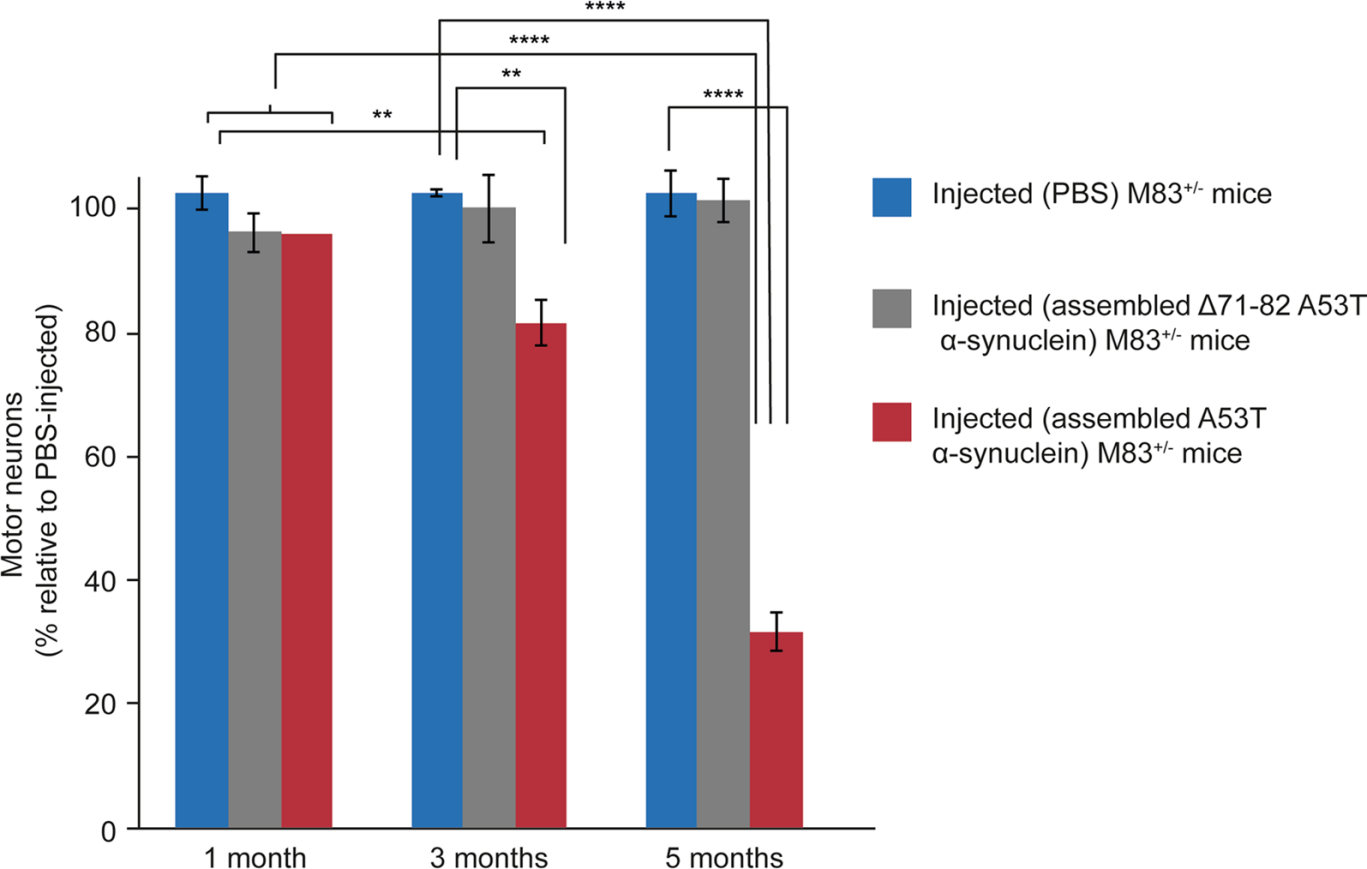
Motor neuron numbers in lumbar spinal cord of M83^±^ mice following intraperitoneal injection of PBS, assembled Δ71–82 A53T α-synuclein and assembled A53T α-synuclein. The number of motor neurons of PBS-injected mice is taken as 100%. Two-way ANOVA F(8,52) = 24.64, followed by Dunnett’s multiple comparisons test. **p < 0.005; ****p < 0.0001 (n = 5)

Mice injected with assembled A53T α-synuclein developed progressive motor impairment and were culled after 155 ± 13 days (n = 13). Controls received an intraperitoneal injection of 200 μl PBS and were culled 165 days later (n = 8). There was no staining for pS129 α-synuclein, nor were there motor symptoms. The effects of assembled A53T α-synuclein on the number of spinal cord motor neurons were compared with those of assembled Δ71–82 A53T alpha-synuclein and PBS (n = 5) (Fig. 5, Additional file 4: Table 1). Mice were culled 1, 3 and 5 months after injection. At 5 months, mice injected with assembled A53T α-synuclein had lost approximately 70% of motor neurons and suffered from hindlimb paralysis. Three months after injection, there was a 20% reduction in the number of motor neurons, with no significant difference from PBS-injected mice after 1 month. The number of motor neurons in Δ71–82 A53T α-synuclein-injected mice did not differ significantly from that of PBS-injected mice.

### Seeded aggregation and motor impairment following injection of brain extract from multiple system atrophy

**Fig. 6:**
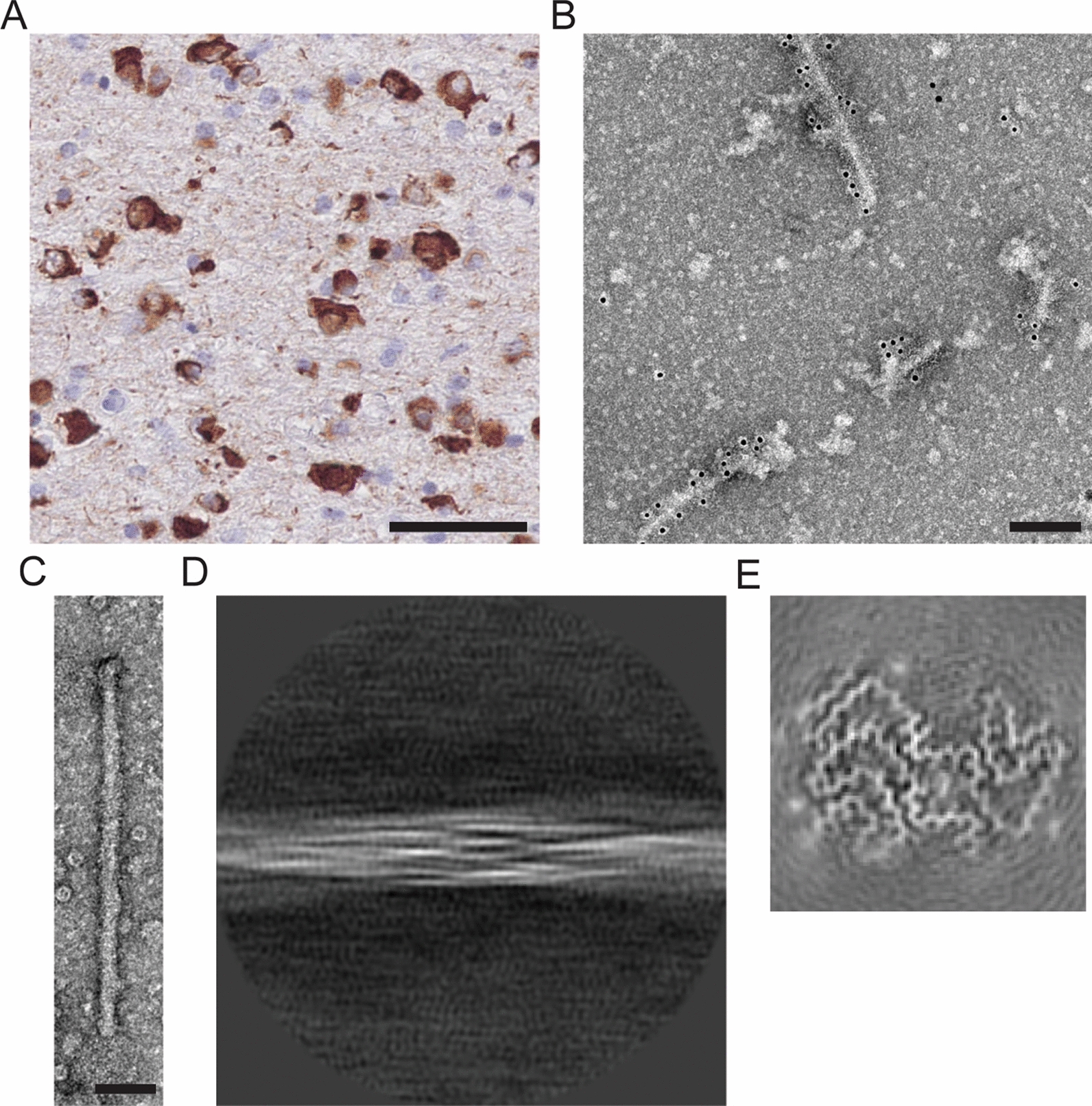
Type II α-synuclein filaments extracted from MSA cerebellum. **A** Staining by ab1903 of abundant neuronal and glial α-synuclein inclusions in cerebellar white matter. Scale bar, 100 μm. **B** Negative-stain immunoelectron microscopy of α-synuclein filaments extracted from MSA cerebellum. Ab59264 was used. Scale bar, 200 nm. **C** Negative-stain electron microscopy of twisted α-synuclein filament. Scale bar, 50 nm. **D** Reference-free 2D class average spanning an entire crossover of filaments extracted from MSA cerebellum. Only type II filaments were present. **E** Cryo-EM map of type II filaments from MSA cerebellum

Cerebellum from a 68-year-old male who had died with a neuropathologically confirmed diagnosis of MSA was used. Numerous α-synuclein-positive glial and neuronal inclusions were present in cerebellar white matter (Fig. 6A). Following sarkosyl extraction and negative staining, α-synuclein filaments were in evidence (Fig. 6B,C). As described [38], they had a diameter of 10 nm and a periodicity of 80–100 nm. Immunoelectron microscopy with PER4 showed the decoration of filaments (Fig. 6B), consistent with previous findings [43]. We imaged sarkosyl-insoluble filaments by cryo-EM. Using reference-free 2D class averaging, we only saw type II filaments (Fig. 6D), as previously reported for cerebellum from another case of MSA [38]. We determined the cryo-EM structures to resolutions sufficient for de novo atomic modelling (Fig. 6E, Table 1). They showed the presence of two protofilaments consisting of residues G14-F94 and G36-Q99. The resolution was 3.27 Å.

**Table 1:** Cryo-EM data collection and processing

Magnification	105,000
Voltage (kV)	300
Electron exposure (e–/Å^2^)	45.0
Defocus range (μm)	−1.8 to −2.4
Pixel size (Å)	1.145
Symmetry imposed	None
Initial particle images (no.)	39,798
Final particle images (no.)	36,247
Map resolution (Å)	3.27
FSC threshold	0.143
Helical twist (°)	−1.36
Helical rise (Å)	4.70

M83^±^ mice were injected intravenously, intraperitoneally and intramuscularly with MSA cerebellar extracts. They developed progressive motor impairment and were culled when exhibiting hindlimb paralysis. Staining for pS129 α-synuclein was present in brain and spinal cord of all cases with a distribution and in amounts similar to those of M83^+/+^ mice with hindlimb paralysis.

For intravenous injection, mice received a daily injection of 100 μl of 100 mg/ml tissue over 4 consecutive days (equivalent to 40 mg tissue). They were culled after 225 ± 19 days (n = 8). For intramuscular injection, mice received a single bilateral injection of 50, 100 or 200 μl of 200 mg/ml tissue (equivalent to 10, 20 or 40 mg tissue) into *gastrocnemius* muscles. They were culled after 238 ± 41 (50 μl, n = 3), 174 ± 36 (100 μl, n = 8) and 146 ± 29 (200 μl, n = 4) days. Control M83^±^ mice were injected with 100 μl cerebellar extract (200 mg/ml) from an age-matched male without synucleinopathy. They were culled after 460 days, when there was neither staining for pS129 α-synuclein nor motor dysfunction.

**Fig. 7:**
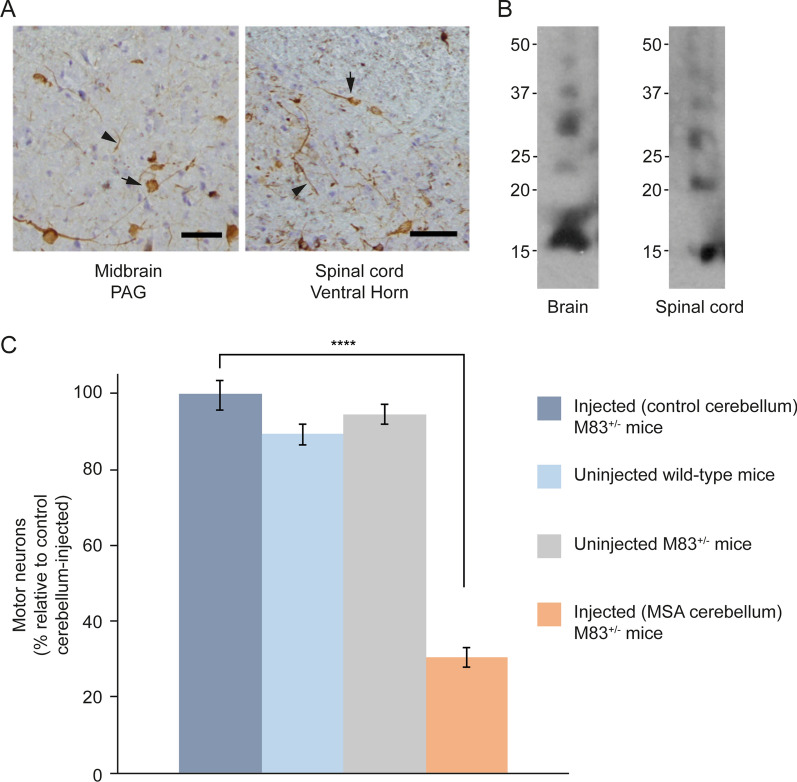
Synucleinopathy in M83^±^ mice following intraperitoneal injection of cerebellar extracts from an individual with neuropathologically confirmed MSA. **A** Staining of midbrain periaqueductal grey (PAG) and lumbar spinal cord (ventral horn) for pS129 α-synuclein (Wako pSyn#64). Arrows denote perikaryal and arrowheads neuritic staining. Scale bars, 50 μm. **B** Immunoblots of sarkosyl-insoluble material from brain and spinal cord using an antibody specific for pS129 α-synuclein (Abcam ab51253). **C** Motor neuron numbers in lumbar spinal cord following intraperitoneal injection of extracts from control and MSA cerebellum. Comparison with uninjected mice. The number of motor neurons of mice injected with extracts from control cerebellum was taken as 100%. One-way ANOVA F(3,16) = 127.5, followed by Tukey’s multiple comparisons test. ****p < 0.0001 (n = 5)

Ten M83^±^ mice were injected intraperitoneally with 100 μl of 200 mg/ml MSA and control cerebellar extracts. Following the injection of MSA extracts, abundant α-synuclein inclusions developed in the central nervous system (Fig. 7A,B, Additional file 2: Figure 2) and the average survival time was 247 ± 55 days. The lifespan of mice injected with control cerebellar extracts was not significantly different from that of uninjected animals and there was no pS129 α-synuclein staining. Five months after the injection of MSA cerebellar extracts, mice had developed hindlimb paralysis and had lost approximately 70% of motor neurons (Fig. 7C, Additional file 5 Table 2). Motor neurons were not lost in age-matched uninjected M83^±^ or control mice.

### Changes in microglial cell morphology accompany neuronal α-synuclein inclusions

**Fig. 8:**
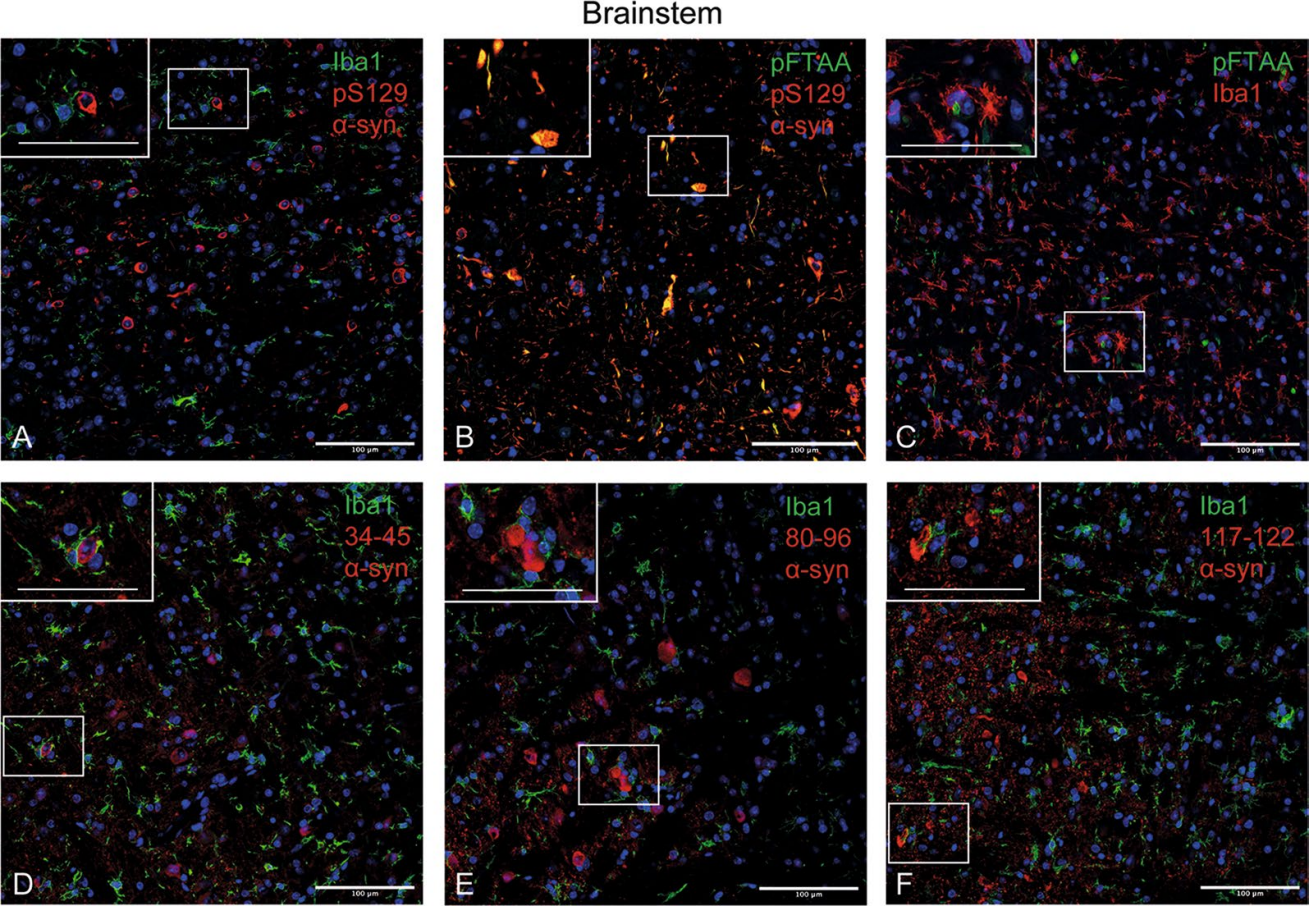
Microglia and α-synuclein inclusions in 20-month-old M83 +/+ mice. **A** Iba1-immunoreactive microglial cells envelop nerve cells immunoreactive with an antibody specific for pS129 α-synuclein (ab184674). **B** Cells stained by pFTAA are also immunoreactive for pS129 α-synuclein (EP1536Y). **C** Iba1-immunoreactive microglial cells envelop nerve cells stained by pFTAA. **D** Iba1-immunoreactive microglial cells envelop nerve cells stained by an antibody specific for residues 34–45 of α-synuclein (α-Syn34-45, BioLegend). **E** Iba1-immunoreactive microglial cells envelop nerve cells stained by an antibody specific for residues 80–96 of α-synuclein (α-Syn80-96, BioLegend). **F** Iba1-immunoreactive microglial cells envelop nerve cells stained by an antibody specific for residues 117–122 of α-synuclein (α-Syn117-122, BioLegend). Scale bars, 100 μm. (019–9741 Wako was used for all Iba1 stainings)

Microglia and nerve cells with α-synuclein inclusions were labelled in brainstem (pons region) from 20-month-old M83^+/+^ mice. Using a panel of epitope-specific α-synuclein antibodies, as well as pFTAA, microglial cells were juxtaposed to nerve cells with α-synuclein inclusions, often with their processes wrapped around these cells. pFTAA-labelled nerve cells were also immunoreactive for pS129 α-synuclein (Fig. 8).

**Fig. 9:**
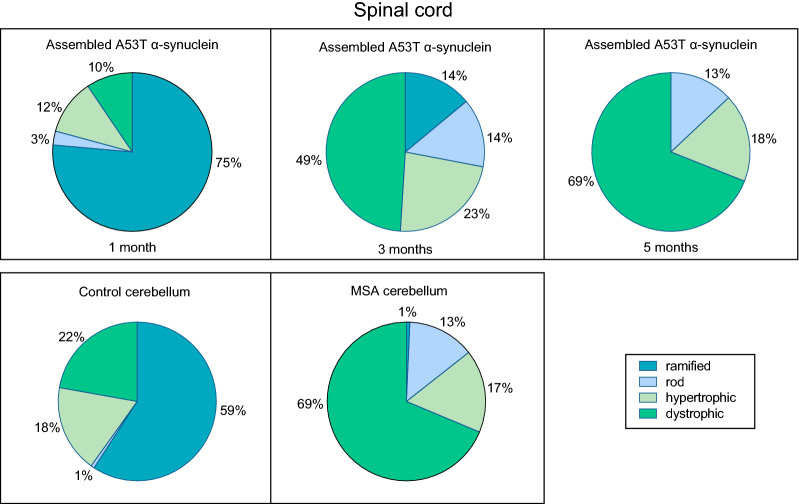
Quantitation of lumbar spinal cord microglial cells of different morphologies in mice injected intraperitoneally with assembled A53T α-synuclein or with MSA brain extract. One month after injection of assembled A53T α-synuclein or control cerebellar extracts, most microglial cells were ramified. At 3 months after injection of assembled A53T α-synuclein, there was an increase in dystrophic microglia. At 5 months after injection of assembled A53T α-synuclein and in end-stage mice injected with MSA cerebellar extracts, dystrophic microglia predominated and ramified microglia were almost absent

Different states of microglia can be defined morphologically [45]. Spinal cord sections from mice injected intraperitoneally with assembled A53T α-synuclein were stained for Iba1 and microglial cell morphologies assessed (Fig. 9, Additional file 3: Figure 3). One month after injection, most microglia were ramified, with a circular cell body and numerous processes extending into the neuropil. By 3 months, they had shifted to a more dystrophic appearance (fivefold increase), with spheroidal, beaded, de-ramified or fragmented processes, as well as to a rod-shaped appearance (threefold increase), with a narrow cell body and a few planar processes. In parallel, a doubling of hypertrophic microglia with short thickened and retracted processes and an enlarged cell body was observed. At 5 months, ramified microglia could no longer be detected, with the proportion of dystrophic microglia continuing to increase. In PBS-injected M83^±^ mice, most microglial cells were ramified at all time-points. When compared to age-matched mice that had been injected with control cerebellar extracts, an approximately threefold increase in dystrophic microglia was observed at end-stage following the injection of cerebellar extracts from a case of MSA.

## Discussion

We show that oral, nasal, intravenous and intraperitoneal administration of assembled A53T α-synuclein induced synucleinopathy in M83^±^ mice. The same was true when cerebellar extracts from a case of MSA with type II α-synuclein filaments were injected intravenously, intramuscularly or intraperitoneally. Synucleinopathy was defined by the presence of abundant immunoreactivity for pS129 α-synuclein in nerve cells and the development of motor impairment, resulting in hindlimb paralysis. Intraperitoneal injection of assembled A53T α-synuclein or cerebellar MSA extracts resulted in a reduction in spinal cord motor neurons.

Following oral and nasal administration of assembled A53T α-synuclein, mice developed hindlimb paralysis. Brains and spinal cords showed abundant pS129 α-synuclein staining and α-synuclein filaments. After oral administration, staining was observed first in the solitary tract nucleus, the dorsal motor nucleus of the vagus nerve and the spinal intermediolateral nucleus, followed by other brain and spinal cord regions. These findings are consistent with early autonomic nervous system involvement, followed by spreading to other regions of the central nervous system. They suggest that oral ingestion of α-synuclein filaments is sufficient for them to cross the epithelial lining of the gastrointestinal tract, before they are taken up by nerve cells in the enteric plexus and reach the brainstem by retrograde trans-synaptic transport along the vagus nerve, consistent with previous findings [25]. Experimental studies in rats have shown that pathological α-synuclein can be transported from the myenteric plexus via the vagus nerve to brain and spinal cord [44, 46].

In addition, our findings indicate that white matter tracts in the brain and spinal cord are affected following the oral administration of assembled α-synuclein, consistent with recent observations that α-synuclein pathology accumulates in afferent sensory tracts, causing degeneration of myelinated fibres, and affects oligodendroglia [23, 24]. This is also consistent with studies in the human brain showing α-synuclein pathology in brainstem fibre tracts [47].

Unlike α-synuclein from wild-type mice, transgenic protein from M83^±^ mice has been reported to be detergent-insoluble [48]. It remains to be seen if α-synuclein seeds can induce pathology over longer time periods in the absence of overexpression, as has been shown for Aβ seeds [49]. Following injection of assembled mouse α-synuclein into the muscle layers of pylorus and duodenum, α-synuclein assembly, loss of dopaminergic neurons and motor impairment were reported in the substantia nigra of non-transgenic mice [50]. Moreover, intravenous injection of assembled human α-synuclein coupled to modified rabies virus glycoprotein resulted in a model of premotor PD in wild-type rats [51].

Nasal administration of assembled A53T α-synuclein also led to abundant inclusions and severe motor impairment in M83^±^ mice. The injection of assembled mouse α-synuclein into the olfactory bulb of wild-type mice has been shown to lead to deficits in olfactory function and the spreading of α-synuclein inclusions to other brain regions [52].

Transport through the blood, followed by crossing of the blood–brain barrier, may be the main route by which assembled α-synuclein reaches the central nervous system following intravenous and intraperitoneal injections, which also gave rise to abundant α-synuclein inclusions and motor impairment. Five months after injection, 70% of spinal cord motor neurons had been lost and abundant α-synuclein inclusions were present, consistent with evidence indicating that filamentous α-synuclein inclusions precede neurodegeneration [53]. These findings confirm previous work, which showed a severe loss of motor neurons in M83^±^ mice following the intramuscular injection of recombinant α-synuclein filaments [13], and extend these findings to intraperitoneal injection. Upon intraperitoneal injection of assembled Δ71–82 A53T α-synuclein, we failed to observe synucleinopathy.

We showed earlier that short filaments of α-synuclein form the majority of seed-competent species in M83^+/+^ brains [35]. Cryo-EM structures identified type I and type II α-synuclein filaments in MSA brains [38] that differ from the structures of assembled recombinant α-synuclein [54–57]. We therefore investigated the effects of MSA cerebellar homogenates with type II α-synuclein filaments. Abundant α-synuclein inclusions and severe motor impairment developed following intravenous, intramuscular and intraperitoneal injections. Five months after intraperitoneal injection of MSA homogenates, approximately 70% of motor neurons had been lost. We thus establish that type II α-synuclein filaments can induce filament formation and neurodegeneration. It remains to be seen if the same is also true of type I filaments. Future studies will have to investigate how the structural differences between α-synuclein filaments from human brain and those assembled using recombinant protein influence prion-like spreading. Protein assemblies extracted from diseased human brains have a greater seeding ability than assemblies of recombinant proteins [58].

When injected intracerebrally into M83^±^ mice, MSA extracts were more potent than PD extracts in inducing synucleinopathy [59–61]. However, the injection of pathological α-synuclein extracted from MSA brains into wild-type mice only induced α-synuclein aggregation in nerve cells [62]. These and other findings [38, 43, 63] suggest that different conformers of assembled α-synuclein may underlie the pathologies of Lewy pathology disorders and MSA. However, despite the fact that the silver-staining properties of PD and MSA inclusions differ, silver staining was like that in PD and M83^+/+^ mice following intracerebral injection of MSA seeds in M83^±^ mice [61]. Taken altogether, our findings indicate that the propagation of α-synuclein inclusions in M83 mice depends not only on the seeds, but also on the levels and properties of transgenically expressed α-synuclein.

Neuroinflammation is a common pathological characteristic of major neurodegenerative diseases [64]. We investigated the relationship between nerve cell inclusions and microglial cells in M83^+/+^ mice. Microglia were juxtaposed to nerve cells with α-synuclein inclusions. Unlike what has been reported previously [65, 66], we failed to observe α-synuclein- or pFTAA-positive inclusions in microglia. Nerve cells labelled by pFTAA were also pS129 α-synuclein-positive. A possible explanation for this discrepancy could be genetic drift in the M83 line, which was bred and maintained in separate colonies. Technical differences cannot be excluded. A shift in microglial cell morphology from a predominantly ramified to a largely dystrophic appearance was detected following intraperitoneal injection of either assembled A53T α-synuclein or cerebellar extract from an MSA patient, indicating a correlation between α-synuclein aggregation and microglial cell morphology. Similar changes in microglial cell morphology have been described in human brains following the development of tau or α-synuclein inclusions [67].

## Conclusion

Upon peripheral administration of assembled A53T α-synuclein or cerebellar homogenates from a case of MSA, we observed a close relationship between the formation of α-synuclein inclusions in nerve cells and neurodegeneration, accompanied by a shift in microglial cell morphology.

## Supplementary Information

**Additional file 1**. **Supplementary Figure 1** pS129 α-Synuclein immunoreactivity in lumbar spinal cord of M83^+/-^ mice following intraperitoneal injection of PBS, assembled Δ71-82 A53T α-synuclein and assembled A53T α-synuclein. pS129 α-Synuclein immunoreactivity of PBS-injected mice is taken as 100%. Two-way ANOVA F(8,60) = 26, *p* < 0.0001, followed by Tukey’s multiple comparisons test. ****p < 0.0001.

**Additional file 2**. **Supplementary Figure 2** pS129 α-Synuclein immunoreactivity in lumbar spinal cord of M83^+/-^ mice following intraperitoneal injection of extracts from control and MSA cerebellum. Uninjected M83^+/-^ mice show comparable pS129 immunoreactivity to those injected with control cerebellum. One-way ANOVA F(2,12) = 64.53, *p* < 0.0001, followed by Tukey’s multiple comparisons test. ****p < 0.0001.

**Additional file 3**. **Supplementary Figure 3** Iba1 immunoreactivity in lumbar spinal cord of M83^+/-^ mice following intraperitoneal injection of assembled A53T α-synuclein. One month post-injection, the vast majority of microglia appeared ramified (black arrows). Three months post-injection, hypertrophic (white arrowhead) and dystrophic (green arrowhead) microglia were also present. Five months post-injection, the majority of microglia appeared dystrophic (green arrowhead). Hypertrophic (white arrowhead) and rod microglia (blue arrowhead) were also present.

**Additional file 4**. **Supplementary Table 1** Motor neuron numbers in lumbar spinal cord of M83^+/-^ mice following intraperitoneal injection of PBS, assembled Δ71-82 A53T α-synuclein and assembled A53T α-synuclein.

**Additional file 5**. **Supplementary Table 2** Motor neuron numbers in lumbar spinal cord of M83^+/-^ mice following intraperitoneal injection of extracts from control and MSA cerebellum. Comparison with uninjected M83^+/-^ mice.

## Data Availability

Data and materials are available from the corresponding author upon request.
