# Supplementary figures and images for: Assembly of α-synuclein and neurodegeneration in the central nervous system of heterozygous M83 mice following the peripheral administration of α-synuclein seeds
Source: Acta Neuropathol Commun. 2021 Nov 24;9:189. doi: 10.1186/s40478-021-01291-7 (PMC8611835; doi:10.1186/s40478-021-01291-7)

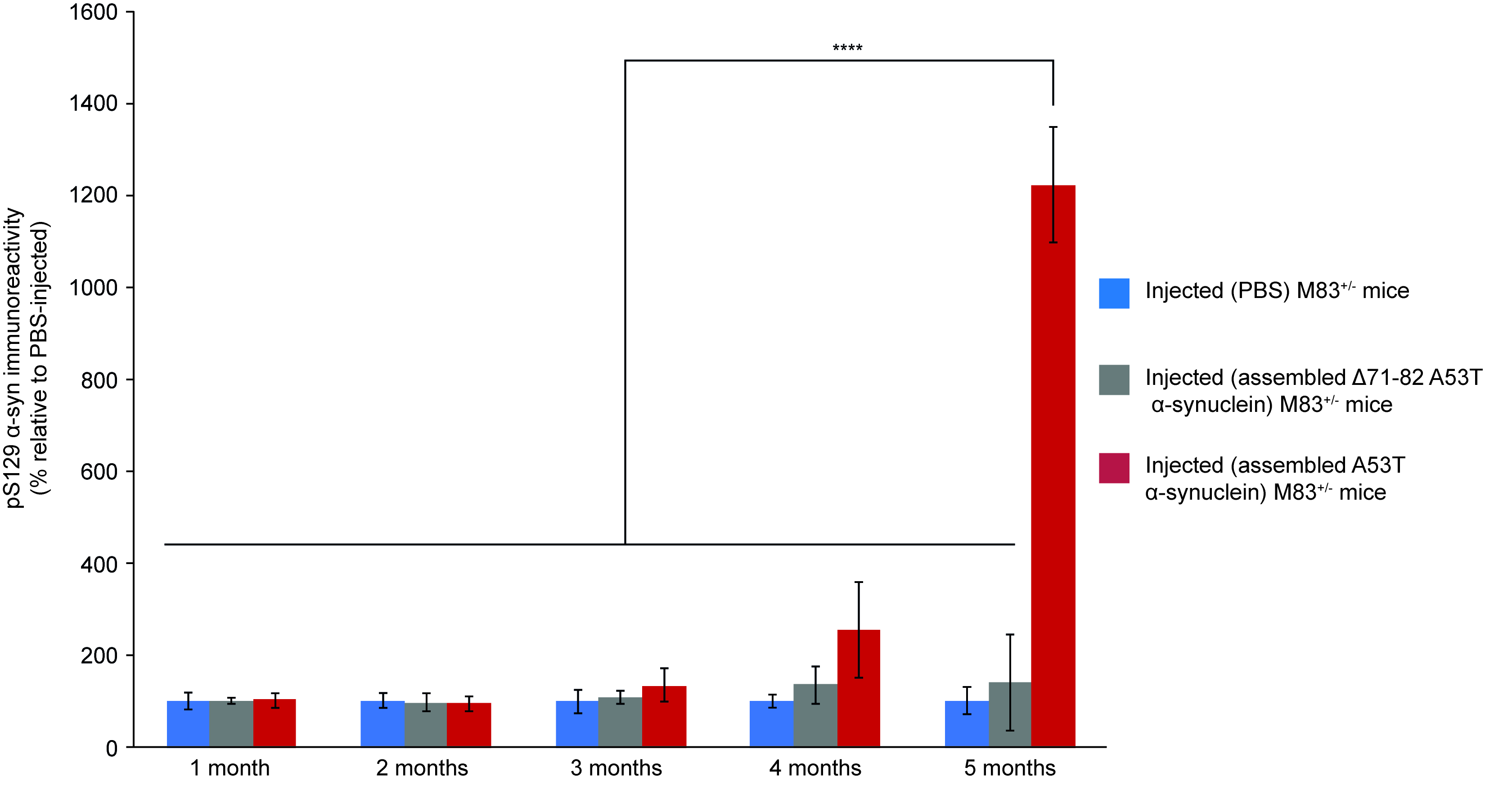

Supplement: Supplementary file 1 — Additional file 1. Supplementary Figure 1 pS129 α-Synuclein immunoreactivity in lumbar spinal cord of M83+/- mice following intraperitoneal injection of PBS, assembled Δ71-82 A53T α-synuclein and assembled A53T α-synuclein. pS129 α-Synuclein immunoreactivity of PBS-injected mice is taken as 100%. Two-way ANOVA F(8,60) = 26, p < 0.0001, followed by Tukey’s multiple comparisons test. ****p < 0.0001. [file 40478_2021_1291_MOESM1_ESM.tif]

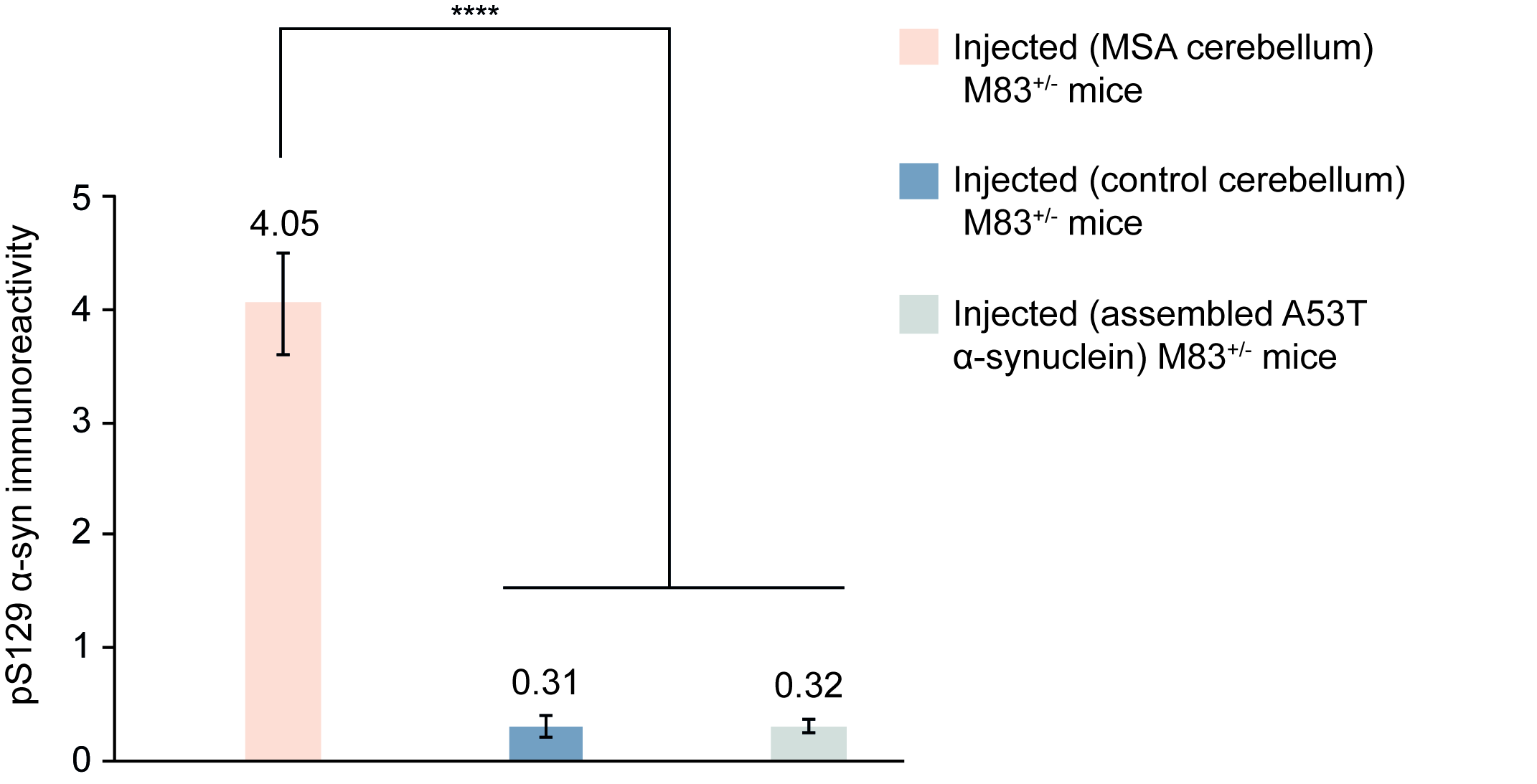

Supplement: Supplementary file 2 — Additional file 2. Supplementary Figure 2 pS129 α-Synuclein immunoreactivity in lumbar spinal cord of M83+/- mice following intraperitoneal injection of extracts from control and MSA cerebellum. Uninjected M83+/- mice show comparable pS129 immunoreactivity to those injected with control cerebellum. One-way ANOVA F(2,12) = 64.53, p < 0.0001, followed by Tukey’s multiple comparisons test. ****p < 0.0001. [file 40478_2021_1291_MOESM2_ESM.tif]

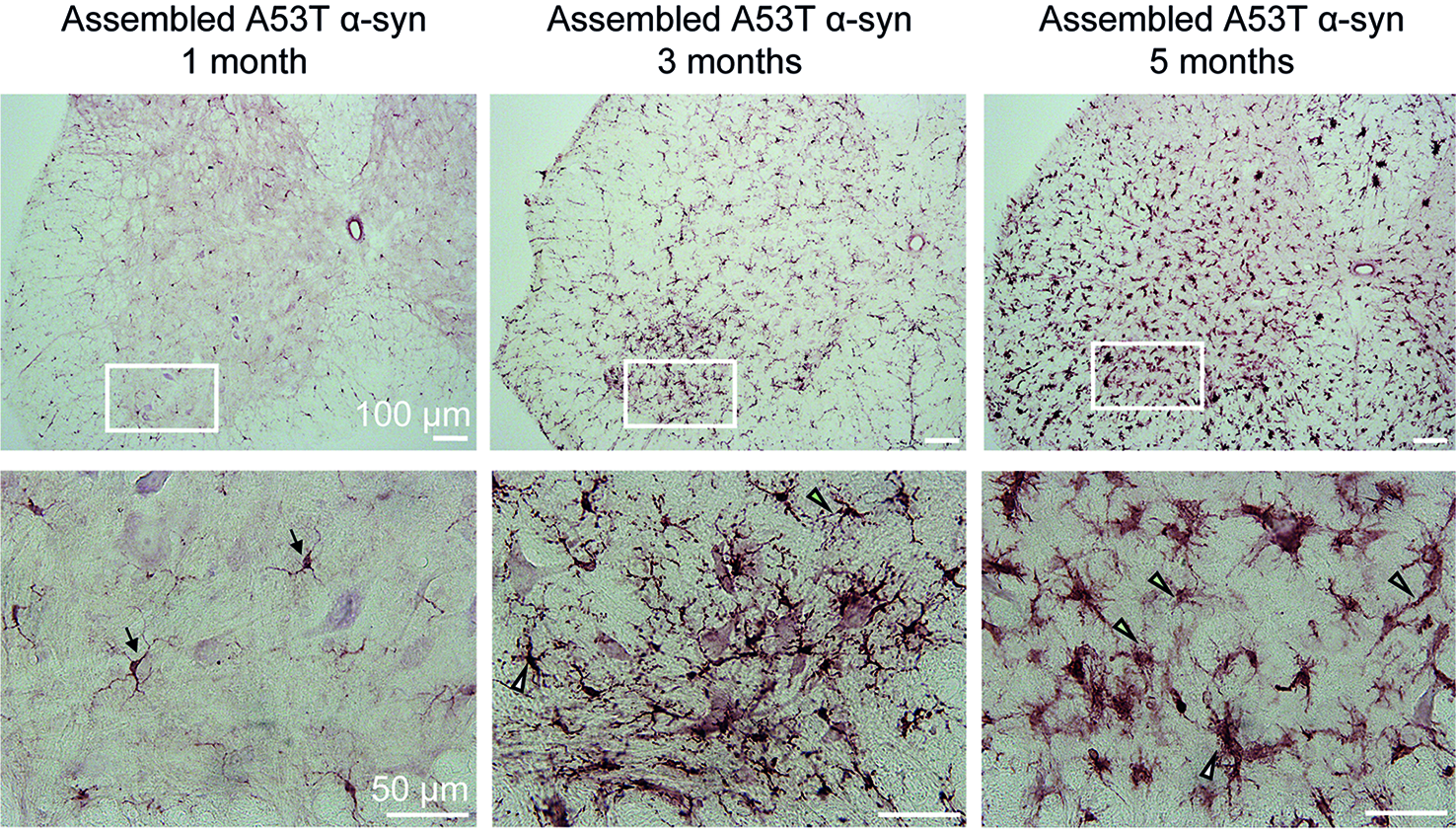

Supplement: Supplementary file 3 — Additional file 3. Supplementary Figure 3 Iba1 immunoreactivity in lumbar spinal cord of M83+/- mice following intraperitoneal injection of assembled A53T α-synuclein. One month post-injection, the vast majority of microglia appeared ramified (black arrows). Three months post-injection, hypertrophic (white arrowhead) and dystrophic (green arrowhead) microglia were also present. Five months post-injection, the majority of microglia appeared dystrophic (green arrowhead). Hypertrophic (white arrowhead) and rod microglia (blue arrowhead) were also present. [file 40478_2021_1291_MOESM3_ESM.tif]
