# Supplementary material for: Assembly of α-synuclein and neurodegeneration in the central nervous system of heterozygous M83 mice following the peripheral administration of α-synuclein seeds
Source: Acta Neuropathol Commun. 2021 Nov 24;9:189. doi: 10.1186/s40478-021-01291-7 (PMC8611835; doi:10.1186/s40478-021-01291-7)
Supplement: Supplementary file 4 — Additional file 4. Supplementary Table 1 Motor neuron numbers in lumbar spinal cord of M83+/- mice following intraperitoneal injection of PBS, assembled Δ71-82 A53T α-synuclein and assembled A53T α-synuclein. [file 40478_2021_1291_MOESM4_ESM.pdf]

Time post-inj	Injected ip with:	n	AVG	SEM	Norm Avg	SEM
1 month	PBS	4	1184.56	43.25	100.00	3.65
1 month	assembled Δ71-82 A53T α-syn	2	1110.84	35.54	93.78	3.00
1 month	assembled A53T α-syn	5	1109.70	43.77	93.68	3.70
2 months	PBS	5	1244.26	34.45	100.00	2.77
2 months	assembled Δ71-82 A53T α-syn	3	1181.95	47.19	94.99	3.79
2 months	assembled A53T α-syn	5	1186.32	35.85	95.34	2.88
3 months	PBS	5	1258.87	37.89	100.00	3.01
3 months	assembled Δ71-82 A53T α-syn	4	1228.29	68.32	97.57	5.43
3 months	assembled A53T α-syn	5	999.76	46.25	79.42	3.67
4 months	PBS	4	1272.03	56.07	100.00	4.41
4 months	assembled Δ71-82 A53T α-syn	4	1296.21	30.32	101.90	2.38
4 months	assembled A53T α-syn	5	845.41	30.50	66.46	2.40
5 months	PBS	5	1310.30	9.51	100.00	0.73
5 months	assembled Δ71-82 A53T α-syn	4	1294.52	44.26	98.80	3.38
5 months	assembled A53T α-syn	5	406.61	41.33	31.03	3.15

8 months old	Uninjected wild-type	5	1282	54.15	97.84	4.13
8 months old	Uninjected $M83^{\pm}$	5	1283.99	24.73	97.99	1.89
