# Supplementary material for: Assembly of α-synuclein and neurodegeneration in the central nervous system of heterozygous M83 mice following the peripheral administration of α-synuclein seeds
Source: Acta Neuropathol Commun. 2021 Nov 24;9:189. doi: 10.1186/s40478-021-01291-7 (PMC8611835; doi:10.1186/s40478-021-01291-7)
Supplement: Supplementary file 5 — Additional file 5. Supplementary Table 2 Motor neuron numbers in lumbar spinal cord of M83+/- mice following intraperitoneal injection of extracts from control and MSA cerebellum. Comparison with uninjected M83+/- mice. [file 40478_2021_1291_MOESM5_ESM.pdf]

Injected ip with:	n	AVG	SEM	Norm Avg	SEM
Injected (control cerebellum) $M83^{\pm}$ mice	5	1403.76	51.51	100.00	3.67
Uninjected wild-type mice	5	1252.83	37.16	89.25	2.65
Uninjected $M83^{\pm}$ mice	5	1325.25	33.40	94.41	2.38
Injected (MSA cerebellum) $M83^{\pm}$ mice	5	427.79	36.36	30.47	2.59
